# Implikationen der prähospitalen Einschätzung des Traumapatienten auf den Behandlungsverlauf – Eine Auswertung aus dem TraumaRegister DGU®

**DOI:** 10.1007/s00101-021-01001-x

**Published:** 2021-07-13

**Authors:** C. Jaekel, L. Oezel, D. Bieler, J. P. Grassmann, C. Rang, R. Lefering, J. Windolf, S. Thelen, Sektion Notfall‑, Intensivmedizin und Schwerverletztenversorgung (Sektion NIS) der DGU

**Affiliations:** 1 Klinik für Orthopädie und Unfallchirurgie, Universitätsklinikum Düsseldorf, Medizinische Fakultät, Heinrich-Heine-Universität Düsseldorf, Moorenstraße 5, 40225 Düsseldorf, Deutschland; 2 Klinik für Unfallchirurgie und Orthopädie, Wiederherstellungs‑, Hand- und Plastische Chirurgie, Verbrennungsmedizin, Bundeswehrzentralkrankenhaus Koblenz, Koblenz, Deutschland; 3 Institut für Forschung in der Operativen Medizin (IFOM), Universität Witten/Herdecke, Köln, Deutschland

**Keywords:** Notfallmedizin, Schwerverletzte, Präklinik, Notarzt, Schockraum, Emergency medical services, Severely injured, Prehospital, Emergency physician, Shock room

## Abstract

**Hintergrund:**

In der prähospitalen Versorgungsphase schwer verletzter Patienten steht die Stabilisierung der Vitalparameter im Vordergrund. Die zügige und möglichst genaue Einschätzung des vorliegenden Verletzungsmusters durch den Notarzt ist entscheidend für die Auswahl der Zielklinik und die Initialbehandlung.

**Ziel der Arbeit:**

Ziel dieser Studie ist es zu eruieren, welchen Einfluss die notärztliche Einschätzung der Verletzungsschwere auf die prähospitale Versorgung und die Schockraumbehandlung hat.

**Material und Methoden:**

Es erfolgt eine Analyse der Daten des TraumaRegister DGU® im Fünfjahreszeitraum von 2015–2019 innerhalb Deutschlands. Die prähospitale notärztliche Einschätzung des Verletzungsmusters wurde anhand des Notarzteinsatzprotokolls erfasst und mit den innerklinischen dokumentierten Diagnosen gemäß den Abbreviated Injury Scale Codes abgeglichen.

**Ergebnisse:**

Insgesamt wurden 47.838 Patienten mit einem durchschnittlichen Injury Severity Score (ISS) von 18,7 Punkten (SA 12,3) eingeschlossen. Zusammenfassend wurden innerklinisch 127.739 verletzte Körperregionen dokumentiert. Von diesen wurden 68,8 % prähospital vom Notarzt richtig vermutet. Somit wurden 31,2 % verletzte Körperregionen nicht detektiert. In insgesamt 42.530 Fällen wurde eine Körperregion als verletzt vermutet, ohne dass sich der Verdacht innerklinisch betätigte. Bei den fehleingeschätzten Verletzungen wurden Schädel-Hirn-Traumata und Gesichtsverletzungen am häufigsten überdiagnostiziert (13,5 % bzw. 14,7 % notärztlich dokumentiert bei nichtvorliegender Diagnose). Thoraxverletzungen wurden am häufigsten unterdokumentiert (17,3 % notärztlich nichtdokumentiert bei abschließend gesicherter Diagnose). Die tatsächliche Gesamtmortalität aller Gruppen entsprach nahezu der erwarteten Mortalität, berechnet mit dem Revised Injury Severity Classification II(RISC II)-Score (12,0 % vs. 11,3 %).

**Diskussion:**

In der prähospitalen Phase der Versorgung von schwer verletzten Patienten wird die durch den Notarzt erfasste Gesamtverletzungsschwere gut eingeschätzt und korreliert mit den eingeleiteten Therapien, der Auswahl der Zielklinik als auch dem innerklinischen Verlauf sowie dem Outcome des Patienten. Die Erfassung von Verletzungen einzelner Körperregionen scheint prähospital jedoch herausfordernd zu sein.

## Abstract (en)

**Background:**

In the prehospital acute treatment phase of severely injured patients, the stabilization of the vital parameters is paramount. The rapid and precise assessment of the injuries by the emergency physician is crucial for the initial treatment and the selection of the receiving hospital.

**Objective:**

The aim of this study was to determine whether the prehospital emergency medical assessment has an influence on prehospital and emergency room treatment.

**Material and methods:**

Data from the TraumaRegister DGU® between 2015 and 2019 in Germany were evaluated. The prehospital emergency medical assessment of the injury pattern and severity was recorded using the emergency physician protocol and compared with the in-hospital documented diagnoses using the abbreviated injury scale.

**Results:**

A total of 47,838 patients with an average injury severity score (ISS) of 18,7 points (SD 12.3) were included. In summary, 127,739 injured body regions were documented in the hospitals. Of these, a total of 87,921 were correctly suspected by the emergency physician Thus, 39,818 injured body regions were not properly documented. In 42,530 cases a region of the body was suspected to be injured without the suspicion being confirmed in the hospital. Traumatic brain injuries and facial injuries were mostly overdiagnosed (13.5% and 14.7%, respectively documented by an emergency physician while the diagnosis was not confirmed in-hospital). Chest injuries were underdocumented (17.3% missed by an emergency physician while the diagnosis was finally confirmed in-hospital). The total mortality of all groups was very close to the expected mortality calculated with the revised injury severity classification II(RISC II)-score (12.0% vs. 11.3%).

**Conclusion:**

In the prehospital care of severely injured patients, the overall injury severity is often correctly recorded by the emergency physician and correlates well with the derived treatment, the selection of the receiving hospital as well as the clinical course and the patient outcome; however, the assessment of injuries of individual body regions seems to be challenging in the prehospital setting.

## Hintergrund

Das optimale Management von schwer verletzten Patienten erfordert eine effiziente prähospitale Behandlung, um eine optimale Versorgung zu gewährleisten. Hierbei sollten das Verletzungsmuster wie auch die Verletzungsschwere möglichst genau eingeschätzt werden, um daraus differenzierte Maßnahmen ableiten zu können. Insbesondere in der Akutphase steht die Stabilisierung der Vitalparameter im Vordergrund, sodass u. U. ein erhöhtes Risiko besteht, Traumafolgen zu übersehen oder fehleinzuschätzen [12]. Ziel der prähospitalen Einschätzung sollte es sein, die zur Verfügung stehenden Ressourcen optimal und zeitgerecht zu nutzen [17]. Hierbei ist zu beachten, dass eine Überschätzung der Verletzungsschwere unnötige Ressourcen verbraucht, während eine Unterschätzung Patienten gefährden kann [28]. Faktoren, welche eine prähospitale Unter- bzw. eine Überschätzung begünstigen, waren bereits Gegenstand verschiedener Studien: Der vorhandene Zeitdruck, die ungünstigeren Untersuchungsbedingungen, die sehr begrenzten diagnostischen Hilfsmittel, der Ausbildungsstand des Notarztes sowie die Qualität der körperlichen Untersuchung können dazu beitragen, klinisch-relevante Verletzungen zu übersehen [6, 20]. Folgen einer etwaigen Fehleinschätzung können in eine unzureichende prähospitale Behandlung als auch zur Wahl einer ungeeigneten primären Zielklinik führen. Dies kann in einer ineffizienten hospitalen Weiterbehandlung münden bzw. zu Weiterverlegungen führen [9]. Somit kann eine zügige und möglichst korrekte notärztliche Ersteinschätzung der relevanten Verletzungen und des Gesamtzustandes des Patienten entscheidend für die Prognose des Schwerverletzten sein. Insgesamt existieren nur wenige Studien, welche die notärztliche Einschätzung der Verletzungsart- und -schwere untersuchen [3, 9, 17]. Esmer et al. konnten in ihrer Studie an 30.777 Patienten insgesamt 51.839 Verletzungen mit einem Abbreviated-Injury-Scale(AIS)-Schweregrad ≥3 identifizieren, von denen insgesamt 71 % korrekt vom Notarzt eingeschätzt wurden. Die Autoren analysierten in diesem Zusammenhang v. a. das jeweilige durch den Notarzt abgeschätzte Verletzungsmuster, ohne die Gesamtverletzungsschwere zu beachten. Auch die daraus abgeleiteten prähospitalen und innerklinischen Maßnahmen sowie letztlich das auf die geschätzte Verletzungsschwere bezogene Outcome wurden von den Autoren nicht untersucht [9].

Ziele der vorliegenden Analyse sind es, die prähospitale Einschätzung des Verletzungsmusters und v. a. auch die Gesamtverletzungsschwere zu beschreiben und mit den tatsächlichen Diagnosen und dem innerklinischen ISS („Injury Severity Score“) zu korrelieren. Hierbei soll der Einfluss der prähospitalen Einschätzung durch den Notarzt auf die Wahl der Zielklinik, die Rettungszeit und die prähospitalen Maßnahmen herausgestellt werden. Zuletzt soll untersucht werden, welchen Einfluss die notärztliche Einschätzung der Verletzungsschwere auf den klinischen Verlauf und das Outcome des Patienten hat.

## Material und Methoden

Das TraumaRegister DGU® der Deutschen Gesellschaft für Unfallchirurgie wurde 1993 gegründet. Ziel dieser multizentrischen Datenbank ist eine pseudonymisierte und standardisierte Dokumentation von Schwerverletzten.

Die Daten werden prospektiv in 4 aufeinanderfolgenden Phasen gesammelt: A) prähospitale Phase, B) Schockraum- und anschließende OP-Phase, C) Intensivstation und D) Entlassung. Die Dokumentation beinhaltet detaillierte Informationen über Demografie, Verletzungsmuster, Komorbiditäten, prähospitales und klinisches Management, intensivmedizinischen Verlauf, wichtige Laborbefunde einschließlich Transfusionsdaten, sowie das Outcome. Einschlusskriterium ist die unfallbedingte Aufnahme in das Krankenhaus über den Schockraum mit anschließender Intensiv- oder IMC-Überwachung oder Ankunft in der Klinik mit Vitalzeichen und Versterben vor Aufnahme auf die Intensivstation.

Die Infrastruktur für Dokumentation, Datenmanagement und Datenanalyse wird von der AUC – Akademie der Unfallchirurgie GmbH, welche der DGU angegliedert ist, bereitgestellt. Die wissenschaftliche Leitung liegt bei der Sektion Notfall‑, Intensivmedizin und Schwerverletztenversorgung der DGU (Sektion NIS). Über eine webbasierte Anwendung geben die teilnehmenden Kliniken ihre pseudonymisierten Daten in eine zentrale Datenbank ein. Wissenschaftliche Auswertungen werden nach einem Reviewverfahren der Sektion NIS genehmigt.

Die teilnehmenden Kliniken sind primär in Deutschland (90 %) lokalisiert, aber eine zunehmende Anzahl von Kliniken aus anderen Ländern trägt ebenfalls Daten bei (zurzeit aus Österreich, Belgien, China, Finnland, Luxemburg, Slowenien, Schweiz, Niederlande und den Vereinigten Arabischen Emiraten). Derzeit fließen jährlich knapp 30.000 Fälle (Basiskollektiv) von über 650 Kliniken in die Datenbank ein.

Die Beteiligung am TraumaRegister DGU® ist freiwillig. Für die dem TraumaNetzwerk DGU® zugehörigen Kliniken ist zumindest die Eingabe eines Basisdatensatzes zur Qualitätssicherung verpflichtend. Etwa die Hälfte aller Fälle wird jedoch mit dem umfangreicheren Standarddatensatz erfasst.

Die vorliegende Arbeit steht in Übereinstimmung mit der Publikationsrichtlinie des TraumaRegister DGU® und ist registriert unter der TR-DGU-Projekt-ID 2020-006.

**Figure 1:**
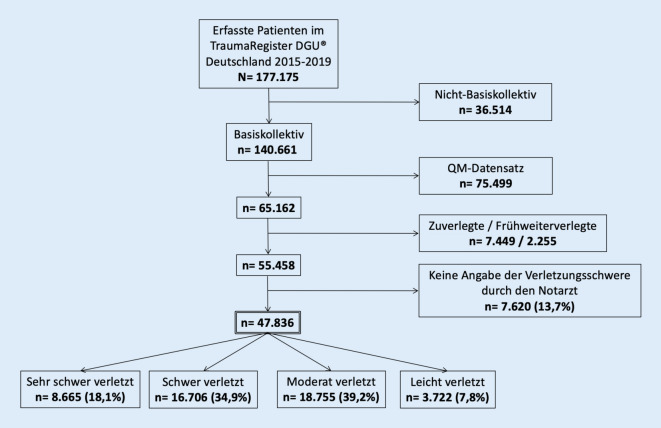

Grundlage der hier vorliegenden Auswertung bildet der Standarddatensatz des TraumaRegister DGU® von 2015–2019 innerhalb Deutschlands (Abb. 1). Eingeschlossen wurde das sog. Basiskollektiv, d. h., alle Patienten mit einem maximalen AIS (MAIS) ≥ 3 sowie Patienten mit einem MAIS = 2, die entweder verstorben sind oder auf der Intensivstation versorgt wurden. Zuverlegte Patienten und früh weiterverlegte (innerhalb von 48 h) Patienten wurden ausgeschlossen. Des Weiteren wurden Patienten, bei denen auf dem Notarztprotokoll keine Angaben zum Verletzungsmuster gemacht wurden, aus der Studie ausgeschlossen. Zur Erfassung der Einschätzung des Verletzungsmusters und der Verletzungsschwere stehen dem Notarzt auf dem Notarztprotokoll 8 verschiedene Körperregionen (Schädel-Hirn, Gesicht, Thorax, Abdomen, Wirbelsäule, Becken, obere Extremität, untere Extremität) zur Verfügung, die sich in 4 Schweregradkategorien einteilen (keine, leichte, mittelschwere und schwere Verletzung). Wurde durch den Notarzt eine Verletzung in einer bestimmten Körperregion dokumentiert und in weiteren Körperregionen nicht, wurden diese als „keine Verletzung“ gewertet. Zur Auswertung der notärztlichen prähospitalen Einschätzungen wurden folgende Gruppen gebildet:

- *sehr schwer verletzt*: mind. 2 Regionen als schwere Verletzung im Notarztprotokoll dokumentiert,
- *schwer verletzt*: mind. eine Region als schwere Verletzung im Notarztprotokoll dokumentiert,
- *moderat verletzt*; mind. eine Region als mittelschwere Verletzung im Notarztprotokoll dokumentiert,
- *leichter verletzt: *nur leichte Verletzungen im Notarztprotokoll dokumentiert.

Zur Erfassung des tatsächlichen, objektiven Verletzungsmusters und dessen Schweregrades wurde die innerklinisch dokumentierte Diagnose entsprechend den AIS-Codes herangezogen. Die gesamten Daten der einzelnen Patienten/Kliniken wurden in der zentralen Datenbank des TraumaRegister DGU® gesammelt und für die vorliegende Auswertung retrospektiv in anonymisierter Form mittels SPSS® (Version 24, IBM Inc., Armonk NY, USA) ausgewertet. Die Darstellung erfolgt mit Fallzahl und Prozenten bzw. Mittelwerten und Standardabweichung (SA). Als primäre Endpunkte wurden Mortalität und Krankenhausliegedauer definiert. Sekundäre Endpunkte waren prähospitale Zeit, Verletzungsschwere mittels ISS, Transportart, Behandlungslevel, Intubation und Reanimation. Als klinisch relevant wurden Zeitunterschiede größer als 5 min und Prozentunterschiede von mindestens 5 % angenommen.

## Ergebnisse

**Figure 2:**
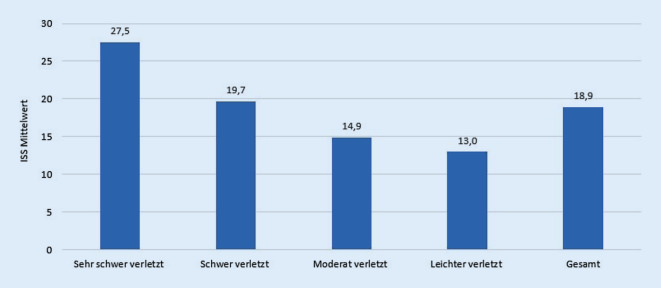

Im Untersuchungszeitraum von 2015–2019 konnten nach den oben genannten Einschlusskriterien insgesamt 47.838 Patienten analysiert werden, mit folgender Verteilung auf die 4 Gruppen gemäß Einschätzung durch den Notarzt (Abb. 2): sehr schwer verletzt: *n* = 8655 (18,1 %); schwer verletzt: *n* = 16.706 (34,9 %); moderat verletzt: *n* = 18.755 (39,2 %); leichter verletzt *n* = 3722 (7,8 %). Das durchschnittliche Alter betrug 51,3 Jahre (SA 22,5; Tab. 1). Die Geschlechterverteilung lag bei 70 % Männern und 30 % Frauen (Tab. 1). Je schwerer die Verletzung durch den Notarzt eingeschätzt wurde, desto mehr lag die Verteilung zugunsten des männlichen Geschlechts. Im Mittel ergab sich ein Injury Severity Score (ISS) von 18,7 Punkten (SA 12,3; Tab. 1). Hierbei ergaben sich für die einzelnen Gruppen folgende ISS-Werte (Abb. 2): sehr schwer verletzt: 27,5 (SA 15,6); schwer verletzt: 19,7 (SA 11,6); moderat verletzt: 14,9 (SA 9,0); leichter verletzt: 13,0 (SA 8,5).

**Table 1:** 

Notärztliche Einschätzung	Sehr schwer verletzt		Schwer verletzt		Moderat verletzt		Leichter verletzt		Gesamt	
	(*n* = 8655)		(*n* = 16.706)		(*n* = 18.755)		(*n* = 3722)		(*n* = 47.838)	
*Allgemeine Daten*										
Alter (Jahre/MW)	48,0	SA 21,3	51,9	SA 22,7	51,8	SA 22,4	53,1	SA 23,5	51,3	SA 22,5
Geschlecht männlich (*n*/%)	6334	73,2 %	11818	70,7 %	12853	68,5 %	2467	66,3 %	33472	70,0 %
ISS (MW)	27,5	SA 15,6	19,7	SA 11,6	14,86	SA 9,0	13,0	SA 8,5	18,7	SA 12,3
Überregionales Traumazentrum (*n*/%)	7481	86,4 %	13899	83,2 %	14350	76,5 %	2545	68,4 %	38275	80,0 %
Regionales Traumazentrum (*n*/%)	1052	12,2 %	2407	14,4 %	3479	18,5 %	912	24,5 %	7850	16,4 %
Lokales Traumazentrum (*n*/%)	122	1,4 %	400	2,4 %	926	4,9 %	265	7,1 %	1713	3,6 %
*Prähospitale Daten*										
Bodengebundener Transport (*n*/%)	5043	59,7 %	11202	68,9 %	14143	77,4 %	3174	87,7 %	33562	72,0 %
Luftgebundener Transport (*n*/%)	3407	40,3 %	5061	31,1 %	4130	22,6 %	446	12,3 %	13044	28,0 %
Zeit bis Klinik (min/MW)	73,6	SA 31,5	72,0	SA 35,2	66,0	SA 30,9	62,5	SA 30,1	69,2	SA 32,7
Bewusstlosigkeit, GCS < 8 (*n*/%)	2902	34,5 %	4527	27,9 %	1102	6,0 %	200	5,6 %	8731	18,8 %
Schock, RR syst < 90 mm Hg (*n*/%)	1573	20,3 %	1363	8,8 %	826	4,7 %	115	3,3 %	3877	8,7 %
Intubation (*n*/%)	4460	51,7 %	6073	36,6 %	1629	8,7 %	178	4,8 %	12340	25,9 %
Reanimation (*n*/%)	842	9,8 %	522	3,1 %	142	0,8 %	30	0,8 %	1536	3,2 %
*Klinische Daten*										
Zeit im SR (min/MW)	75,3	SA 68,3	70,7	SA 62,5	72,4	SA 61,1	76,2	SA 65,5	72,6	SA 63,3
Zeit bis CT (min/MW)	21,4	SA 15,1	19,9	SA 13,2	20,1	SA 14,7	24,1	SA 19,8	20,9	SA 14,8
ICU-Liegetage (MW)	10,7	SA 15,0	7,2	SA 10,3	4,2	SA 7,3	3,4	SA 6,2	6,4	SA 10,4
Krankenhausliegetage (MW)	21,4	SA 21,8	17,4	SA 18,3	12,8	SA 12,8	9,9	SA 10,2	15,7	SA 17,0
Frühmortalität, erste 24 h (*n*/%)	1144	13,2 %	1455	8,7 %	321	1,7 %	54	1,5 %	2974	6,2 %
Gesamtletalität (*n*/%)	1826	21,1 %	2767	16,6 %	956	5,1 %	183	4,9 %	5732	12,0 %
Letalitätsprognose gemäß RISC II	–	21,1 %	–	15,0 %	–	4,7 %	–	4,9 %	–	11,3 %

*MW* Mittelwert, *SA* Standardabweichung, *ISS* Injury Severity Score, *GCS* Glasgow Coma Scale, *SR* Schockraum, *CT* Computertomographie, *ICU* Intensivstation, *RISC II* Revised Injury Severity Classification II

### Tatsächliches Verletzungsmuster vs. prähospitale Einschätzung des Verletzungsmusters

**Figure 3:**
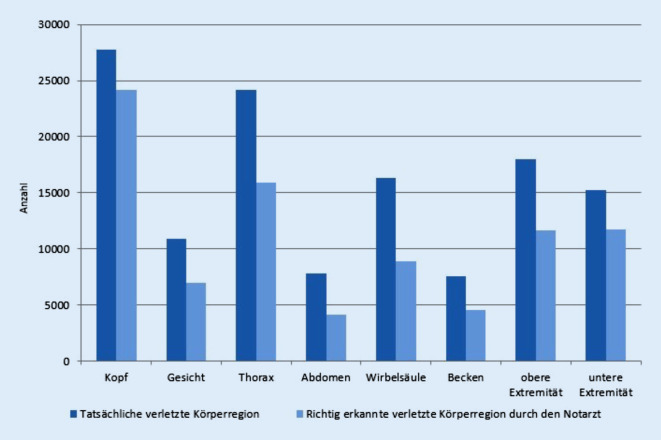

Bei dem untersuchten Patientenkollektiv wurden insgesamt 127.739 verletzte Körperregionen innerklinisch diagnostiziert (Abb. 3). Schädel-Hirn-Traumata waren mit 21,7 % die am häufigsten dokumentierten Traumafolge, gefolgt von Thoraxverletzungen (18,9 %). Beckenverletzungen (5,9 %) und Verletzungen des Abdomens (6,1 %) traten am seltensten auf. Von allen verletzten Körperregionen wurden insgesamt 68,8 % auch vom Notarzt prähospital vermutet (Abb. 3). Somit wurden insgesamt 31,2 % verletzte Körperregionen nicht detektiert. Mit *n* = 30.624 war das Schädel-Hirn-Trauma die am häufigsten gestellte Verdachtsdiagnose. Abdominelle Verletzungen (*n* = 9381) wurden am seltensten vermutet. Bezogen auf die verschiedenen Körperregionen wurden somit 70,2–83,2 % aller Verletzungen (richtig-positiv und richtig-negativ) auch als Verdachtsdiagnose durch den Notarzt vermutet. Besonders Verletzungen des Beckens, des Abdomens und der unteren Extremität wurden korrekt eingeschätzt (alle > 80 %; Tab. 2). Thoraxverletzungen wurden in 17,3 % im Notarztprotokoll nicht dokumentiert, trotz vorliegender Verletzung (Tab. 2, nicht erkannt). Des Weiteren waren 15,6 % der Verletzungen der Wirbelsäule nicht als prähospitale Verdachtsdiagnose angegeben. Verletzungen im Gesicht wurden zu 14,7 % als Verdachtsdiagnose dokumentiert, ohne sich zu bestätigen, und waren somit überschätzt. Insgesamt wurde in 42.530 Fällen eine Körperregion als verletzt vermutet, ohne dass sich der Verdacht innerklinisch betätigte.

**Table 2:** 

Region	Verletzung vorliegend	Prävalenz (%)	Nicht erkannt (Falsch-negativ) (%)	Überschätzung (Falsch-positiv) (%)	Summer abweichender Einschätzungen (%)
Thorax	24.170	50,5	17,3	8,1	25,4
Wirbelsäule	16.332	34,1	15,6	11,7	27,3
Obere Extremität	18.023	37,7	13,3	8,8	22,1
Gesicht	10.918	22,8	8,2	14,7	22,9
Abdomen	7791	16,3	7,7	11,0	18,7
Kopf	27.732	58,0	7,4	13,5	20,9
Untere Extremität	15.201	31,8	7,3	9,5	16,8
Becken	7572	15,8	6,3	11,6	17,9

### Prähospitale Maßnahmen

**Figure 4:**
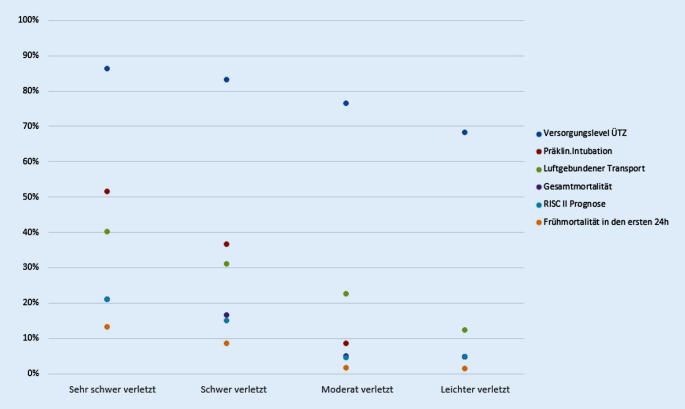

Bei 80 % aller analysierten Patienten erfolgte die primäre Zuweisung in ein überregionales Traumazentrum (Tab. 1). Je leichter verletzt die Patienten durch den Notarzt eingeschätzt wurden, desto seltener wurde ein überregionales Traumazentrum angefahren (Abb. 4). Zu beachten ist, dass weiterhin 68,4 % aller leichter verletzt eingeschätzten Patienten dem Maximalversorger zugewiesen worden sind. Dem gegenüber sind als leichter verletzt eingeschätzte Patienten nur in 7,1 % der Fälle in einem lokalen Traumazentrum zugewiesen worden. Wurde das Verletzungsausmaß schwerer eingestuft, wurde häufiger ein luftgebundener Transport in die Klinik durchgeführt (Tab. 1; Abb. 4).

Je ausgeprägter das Verletzungsmuster des Patienten durch den Notarzt eingestuft wurde, desto länger dauerte die prähospitale Versorgungszeit bis zum Eintreffen in der Klinik (Tab. 1). Im Mittel lag diese bei 69,2 min (SA 32,7). Zusammengefasst war der Anteil an Patienten, welche prähospital bewusstlos (34,5 %) oder im Schock (20,3 %) waren, in der Gruppe, welche als am schwersten verletzt eingestuft worden war, am höchsten (Tab. 1). Ebenso wurde diese Patientengruppe am häufigsten intubiert (51,75 %) und reanimiert (9,8 %). Als leichter verletzt eingeschätzte Patienten musste in 4,8 % der Fälle intubiert und in 0,8 % der Fälle reanimiert werden (Tab. 1; Abb. 4).

### Innerklinischer Verlauf und Outcome

**Figure 5:**
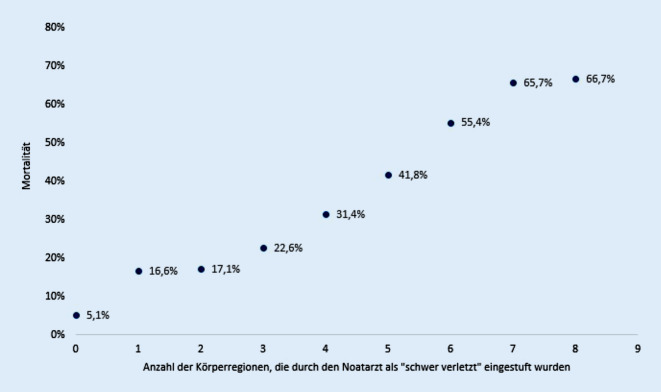

Sowohl die Zeit für die Schockraumversorgung als auch die Zeit bis zur Durchführung des CT sinkt mit zunehmender Einschätzung der Verletzungsschwere zwischen den Gruppen leichter verletzt, moderat verletzt und schwer verletzt, steigt jedoch bei den sehr schwer Verletzten wieder an (Tab. 1). Die durchschnittliche Krankhausliegedauer aller Patienten lag bei 15,7 Tagen (SA 17,0) mit der Notwendigkeit einer intensivmedizinischen Behandlung von durchschnittlich 6,4 Tagen (SA 10,4; Tab. 1). Die Frühmortalität und auch die Gesamtmortalität steigen korrelierend mit zunehmender angenommener Verletzungsschwere (Abb. 5). Die über den RISC II Score berechnete Letalitätsprognose deckt sich für die einzelnen Gruppen sehr genau (Tab. 1; Abb. 4). Unterteilt man die Gruppe der sehr schwer Verletzten weiter anhand der Zunahme der als schwer verletzt eingeschätzten Körperregionen, ist eine stetige Zunahme der Gesamtmortalität zu beobachten (Abb. 5). Des Weiteren wurde die Mortalität bestimmter Untergruppen untersucht. Insgesamt 522 Patienten, die durch den Notarzt als schwer verletzt (*n* = 400) oder sehr schwer verletzt (*n* = 122) eingestuft wurden, sind trotz des Verletzungsmusters einem lokalen Traumazentrum zugewiesen worden. Von diesen Patienten sind 9,6 % verstorben (95 %-Konfidenzintervall 7,1–12,1). Verglichen mit dem prognostizierten Letalitätsrisiko gemäß RISC II Score war ein Wert von 8,6 % vorhergesagt worden. Hieraus ergibt sich kein signifikanter Unterschied. Der ISS dieser 522 Patienten lag bei 14,7 Punkten und war somit signifikant geringer als der durchschnittliche ISS der schwer (19,7 Punkte) und sehr schwer (27,5 Punkte) verletzten Patientengruppen (*p* < 0,001). Insgesamt wurden 2545 der leichter verletzt eingeschätzten Patienten in ein überregionales Traumazentrum eingeliefert, welche eine Gesamtmortalität von 5,5 % aufwiesen. Dieses deckt sich mit dem über den RISC II Score berechneten Wert von 5,1 %.

## Diskussion

Die prähospitale Einschätzung sowohl des Verletzungsmusters als auch der Verletzungsschwere von schwerstverletzten Patienten durch den Notarzt ist herausfordernd und von vielen Faktoren abhängig. Die vorliegende Auswertung bestätigt, dass die durch den Notarzt erhobenen Verdachtsdiagnosen nicht selten von den tatsächlichen Diagnosen abweichen. Insbesondere die innerklinische Erstversorgung von schwer verletzten Patienten im Rahmen der Schockraumbehandlung wurde in den letzten Jahrzehnten vielfach diskutiert und zunehmend standardisiert. Es konnte gezeigt werden, dass die Einführung von problem- und prioritätenorientierten Behandlungsalgorithmen den zeitlichen Ablauf sowohl diagnostischer als auch therapeutischer Maßnahmen verkürzt und in der Folge auch die Mortalität reduziert [21]. Auch das prähospitale Setting wurde in den letzten Jahren zunehmend standardisiert. Es werden auch hier bestimmte Algorithmen als erforderlich betrachtet, um wertvolle Zeit zu gewinnen und das Outcome des Patienten zu verbessern [26]. Schweiberer et al. fassten bereits 1987 die Kombination folgender Fähigkeiten im Rahmen der Schwerstverletztenversorgung als essenziell zusammen: das rasche Erfassen der traumatischen Gesamtbelastung, das zügige Erkennen aller bedrohlichen und relevanten Verletzungsmuster sowie das schnelle Setzen der richtigen Prioritäten [22]. Zusammengefasst haben sich hieraus standardisierte Verfahren zur prähospitalen Versorgung von schwer verletzten Patienten etabliert (z. B.: Prehospital-Trauma-Life-Support(PHTLS)-Algorithmus, Europäischer Trainingskurs zur erweiterten prähospitalen Traumaversorgung), die dabei helfen können, ein prioritätenorientiertes, effektives prähospitales Traumamanagement zu gewährleisten [1, 30]. Nichtsdestotrotz wird die prähospitale Versorgung durch verschiedenste Bedingungen erschwert, was in Fehleinschätzungen münden kann.

### Verletzungsmuster und Verletzungsschwere

Beim schwer verletzten Patienten stellt das Thoraxtrauma die dritthäufigste Todesursache nach dem Schädel-Hirn-Trauma und den abdominellen Verletzungen dar [7, 13]. In der vorliegenden Studie weisen 50,5 % der untersuchten Patienten eine Thoraxverletzung auf. Dieses steht im Einklang mit der vorliegenden Literatur, die in nahezu 50 % aller schwerstverletzten Patienten eine Thoraxverletzung nachweist [11, 27]. In dem hier untersuchten Patientenkollektiv war in 17,3 % kein Verdacht einer Thoraxverletzung gestellt worden, obwohl sich eine solche im weiteren Verlauf herausstellte. Umgekehrt wurde in 8,1 % der Fälle ein Verdacht einer Thoraxverletzung durch den Notarzt dokumentiert, ohne dass eine Verletzung in den abschließenden Diagnosen angegeben wurde. Thoraxverletzungen scheinen eine große Herausforderung an den Notarzt zu stellen. Mit der prähospitalen Sonographie steht ein Diagnostikum zur Detektion möglicher Thoraxverletzungen zur Verfügung. Die Sonographie des Thorax ist insbesondere zur Detektion von traumatischen Pneumothoraces und Hämatothoraces ein gutes Instrument [24]. Letztlich sollte jedoch das Ziel der prähospitalen Sonographie nicht die Vorwegnahme einer Schockraumdiagnostik sein. Sie sollte aus Sicht der Autoren vielmehr als variables „adjunct“ dazu dienen, die Indikation für die Notwendigkeit einer etwaigen prähospitalen Intervention zu erhärten bzw. zu bestätigen. Eine Sonographie ohne therapeutische Konsequenz kann u. U. wertvolle Zeit kosten. Die bisherige Literatur gibt diesbezüglich jedoch kaum eine Evidenz wieder, die einen positiven Einfluss auf das Management und letztlich auch das Outcome des Schwerverletzten zeigt [18].

Nach den Thoraxverletzungen werden Wirbelsäulenverletzungen im vorliegenden Patientenkollektiv am zweithäufigsten nicht erfasst. Sie wurden in 15,6 % durch den Notarzt nicht dokumentiert, nach innerklinischem Verlauf aber festgestellt. Vorherige Studien konnten bereits zeigen, dass insbesondere Verletzungen der Halswirbelsäule, bei bewusstlosen und verwirrten Patienten problematisch sind. Des Weiteren konnten das Vorliegen von multiplen Verletzungen bei Traumapatienten mit einem ISS ≥16 als auch ein GCS ≤8 als Prädiktoren für übersehene spinale Verletzungen eruiert werden [8, 10]. In diesem Hinblick ist besonders zu beachten, dass spinale Verletzungen häufig assoziiert mit Schädel-Hirn-Traumata auftreten [19].

Neben den einzelnen Verletzungsmustern sollte aber insbesondere auch die gesamte Verletzungsschwere betrachtet werden. Der ISS ist hierbei ein gutes Instrument, die Verletzungsschwere eines Traumapatienten abzuschätzen. Zusammengefasst schätzt der Notarzt die Verletzungsschwere der Patienten, verglichen mit dem tatsächlichen ISS, gut ein (Abb. 2). So korreliert der innerklinisch bestimmte ISS der Traumapatienten mit der notärztlichen Einschätzung von leichter (13,0 Punkte) über moderat (14,9 Punkte) bis schwer (19,7 Punkte) und sehr schwer (27,5 Punkte) verletzt. Hieraus abgeleitet können dann durch den Notarzt auch entsprechend die weiteren prähospitalen Maßnahmen bis hin die Wahl zur richtigen Zielklinik getroffen werden.

### Prähospitale Maßnahmen und Zielklinik

Die Abhängigkeit von der Prognose und dem Outcome eines schwerstverletzten Patienten von prähospital durchgeführten, teils invasiven Maßnahmen ist ein häufiges Thema wissenschaftlicher Arbeiten [4]. Die vorliegende Auswertung zeigt, je schwerer das Verletzungsmuster des Patienten durch den Notarzt eingestuft wurde, desto länger dauerte die prähospitale Versorgungszeit bis zum Eintreffen in der Klinik. Diese lag im Mittel bei 69,2 min (SA 32,7). Bisherige Studien belegen, dass das Outcome eines schwer verletzten Patienten sowohl von einer „adäquaten“ präklinischen Versorgung als auch dem zügigen Transport in ein geeignetes Traumazentrum abhängt [5, 16, 23, 25]. Die „adäquate“ präklinische Versorgung samt ihren Maßnahmen kann hierbei jedoch nicht allgemeingültig formuliert werden. Insgesamt kann eine schnelle und strukturierte Durchführung der notwendigen Maßnahmen am Unfallort die Zeit bis zur Zielklinik reduzieren und die klinische Behandlung des Schwerverletzten optimieren.

Die Abhängigkeit des Outcomes eines schwer verletzten Patienten vom Versorgungslevel des erstangefahrenen Krankenhauses war ebenfalls Gegenstand vorheriger Studien. So konnte im US-amerikanischen Raum gezeigt werden, dass die Case-Mix-adjustierte Mortalität von 18 Level-1-US-Traumazentren, verglichen mit 51 anderen US-Kliniken, signifikant verringert war [15]. Demgegenüber konnten Bieler et al. anhand der Daten von 2002–2011 aus dem TraumaRegister DGU® darstellen, dass Patienten, welche in Level-II- und Level-III-Traumazentren versorgt wurden, gegenüber Patienten, welche in Level-I-Traumazentren gebracht wurden, keine signifikanten Unterschiede in der Mortalitätsrate aufweisen [5]. In den hier vorliegenden Daten kann gezeigt werden, dass die Einschätzung der Verletzungsschwere einen Einfluss auf die Zielklinik hat und schwerstverletzte Patienten häufiger dem überregionalen Traumazentrum zugewiesen werden als weniger schwer verletzte Patienten (Abb. 4). Nicht nur die Auswahl der Zielklinik, auch die Wahl des Rettungsmittels ist unmittelbar abhängig von der notärztlichen eingeschätzten Verletzungsschwere (Abb. 4). Je schwerer das Verletzungsmuster eines Patienten eingestuft wurde, desto häufiger wurde ein luftgebundener Transport durchgeführt. Patienten, welche als sehr schwer verletzt eingestuft wurden und im Durchschnitt einen ISS von 27,5 Punkten aufwiesen, wurden in 40,3 % luftgebunden transportiert. Vermutete leichter verletzte Patienten mit einem durchschnittlichen ISS von 13,0 Punkten wurden nur in 12,3 % mittels Rettungshubschrauber der Zielklinik zugewiesen. Weinlich et al. konnten zeigen, dass durch einen luftgebundenen Transport eine signifikante Verbesserung sowohl der innerklinischen Mortalität als auch des Outcomes, gemessen an der Glasgow Outcome Scale (GOS), erzielt werden kann [29]. Die Autoren stellen aber gerade in Deutschland eine hohe Rate (44 %) an Nachalarmierungen des Rettungshubschraubers und somit Verzögerungen im präklinischen Ablauf fest, sodass sie eine klare Definition von Kriterien zur direkten Alarmierung eines Rettungshubschraubers fordern. Weitergehend analysierten Andruszkow et al., welche ebenfalls einen Überlebensvorteil für luftgebunden transportierte Traumapatienten aufzeigen konnten, Subgruppen, die besonders von einem Rettungshubschraubertransport profitieren [2]. Neben Patienten höheren Alters (> 55 Jahre) sind interessanterweise die weniger schwer verletzten Patienten (ISS: 9–15) diejenigen, die einen Überlebensvorteil aufzeigen.

### Outcome

Anhand des RISC II Score kann zuverlässig mittels 14 verschiedener Parameter die erwartete Mortalität eines Patientenkollektivs bestimmt werden [14]. Zwischen der erwarteten Mortalität und der tatsächlichen Gesamtmortalität der einzelnen Gruppen zeigt sich in den hier vorliegenden Daten eine gute Übereinstimmung, resp. zeigt sich eine deutliche Korrelation der notärztlichen Einschätzung hinsichtlich der Gesamtverletzungsschwere (Abb. 4). Des Weiteren kann bei näherer Betrachtung der Gruppe sehr schwer verletzter Patienten eine Linearität der Sterblichkeitszunahme mit der Anzahl an dokumentierten schweren Verletzungen verzeichnet werden (Abb. 5). Vor diesem Hintergrund sollte der prähospitalen Einschätzung des Notarztes im Rahmen der telefonischen Schockraumalarmierung unbedingt hohe Aufmerksamkeit geschenkt werden. Ob die notärztliche Einschätzung allerdings auf den weiteren innerklinischen Verlauf eine kausale Auswirkung hat, ist anhand der hier vorliegenden retrospektiven Studie nicht beantwortbar.

### Limitationen

Bei der vorliegenden Studie handelt es sich um eine retrospektive Datenauswertung, der auf dem Notarztprotokoll erfassten Verdachtsdiagnosen und deren Verletzungsschwere. Hierbei wird die Einschätzung durch den Notarzt anhand von 4 Schweregradkategorien erfasst: „keine“, „leicht“, „mittelschwer“ und „schwer“. Auch wenn im Rahmen der prähospitalen Versorgung eine richtige Verdachtsdiagnose gestellt wurde, kann diese aufgrund der individuell sehr unterschiedlichen Interpretation dieser Kategorien im Schweregrad unterschiedlich bewertet worden sein. In 13,7 % aller untersuchten Fälle war durch den Notarzt keinerlei Verletzung dokumentiert worden, sodass diese Fälle von der Auswertung ausgeschlossen werden mussten (durchschnittlicher ISS dieser Patienten: 18,9). Auch wenn sich die zur Datenauswertung gebildeten Gruppen hinsichtlich des durchschnittlichen ISS relativ homogen verteilen, wurden diese Gruppen willkürlich gebildet.

Weiterhin muss beachtet werden, dass viele Einflussfaktoren eine nicht zu unterschätzende Rolle bei der Bewertung der Ergebnisse spielen. Insbesondere das Outcome des Schwerstverletzten unterliegt einer Vielzahl von Faktoren (z. B. Erfahrung des Rettungsdienstpersonals, Uhrzeit und Ort des Traumas, Rettungsmittel, versorgende Einrichtung, Patientenfaktoren), die nur unzureichend in ihrer Gesamtheit erfasst werden können. Eine Differenzierung zwischen urbaner bzw. ländlicher präklinischer Versorgung erlaubten die in dieser Studie analysierten Daten nicht. Auch die Entfernung vom Einsatzort zur Zielklinik ist nicht Gegenstand der Untersuchung, allerdings spielt die jeweilige Transportzeit in der prähospitalen Entscheidungsfindung für den Notarzt eine Rolle. So könnte beim instabilen Patienten mit Abdominaltrauma die Entscheidung für den Transport in ein nahegelegenes Traumazentrum mit dem Ziel der Laparotomie getroffen werden, bei gleichem Einsatzort und dem Vorliegen eines schweren Schädel-Hirn-Traumas der Transport in ein überregionales Traumazentrum. Dies stellt aus unserer Sicht zumindest eine Limitation dar, die erwähnt werden muss, da die Daten die Frage nicht klären können.

## Fazit für die Praxis

- Die Gesamtverletzungsschwere wird durch den Notarzt überwiegend gut eingeschätzt und korreliert deutlich mit den abgeleiteten Therapien, der Auswahl der Zielklinik als auch dem innerklinischen Verlauf.
- Für die Primärversorgung im Schockraum bleibt jedoch festzuhalten, dass mehr als jede sechste Thoraxverletzung nicht als solche angekündigt wurde.
- Die vorliegende Arbeit zeigt insbesondere eine deutliche Korrelation der notärztlichen Einschätzung der Gesamtverletzungsschwere mit der beobachteten Mortalität.
- Vor diesem Hintergrund sollte der prähospitalen Anmeldung des Notarztes für eine Schockraumalarmierung hohe Priorität eingeräumt und diese nicht infrage gestellt werden.
